# Phase-Field Study of Exchange Coupling in Co-Pt Nonstandard Nanochessboards

**DOI:** 10.3390/ma16165689

**Published:** 2023-08-18

**Authors:** Keran Xu, Jiabei Tang, Yanzhe Wang, Yinning Zhu, Liwei D. Geng

**Affiliations:** Department of Materials Science and Engineering, Sichuan University-Pittsburgh Institute, Sichuan University, Chengdu 610065, China; 2020141520151@stu.scu.edu.cn (K.X.); 2020141520201@stu.scu.edu.cn (J.T.); 2020141520181@stu.scu.edu.cn (Y.W.); 2020141520019@stu.scu.edu.cn (Y.Z.)

**Keywords:** exchange coupling, phase-field modeling, nanochessboard, domain mechanism

## Abstract

The Co-Pt binary system can form a two-phase nanochessboard structure comprising regularly aligned nanorods of magnetically hard tetragonal L1_0_ phase and magnetically soft cubic L1_2_ phase. This Co-Pt nanochessboard, being an exchange-coupled magnetic nanocomposite, exhibits a strong effect on magnetic domains and coercivity. While the ideal nanochessboard structure has tiles with equal edge lengths (a = b), the non-ideal or nonstandard nanochessboard structure has tiles with unequal edge lengths (a ≠ b). In this study, we employed phase-field modeling and computer simulation to systematically investigate the exchange coupling effect on magnetic properties in nonstandard nanochessboards. The simulations reveal that coercivity is dependent on the length scale, with magnetic hardening occurring below the critical exchange length, followed by magnetic softening above the critical exchange length, similar to the standard nanochessboards. Moreover, the presence of unequal edge lengths induces an anisotropic exchange coupling and shifts the coercivity peak with the length scale.

## 1. Introduction

**Figure 1:**
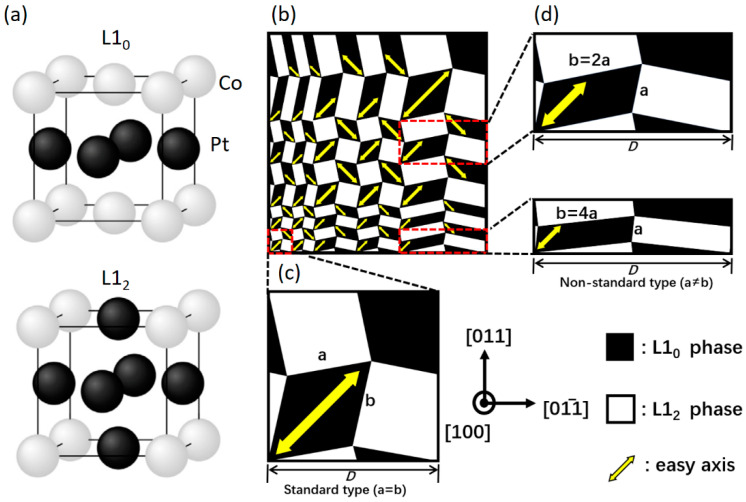
Illustration of (**a**) L1_0_ and L1_2_ atomic structures and (**b**) typical nanochessboard structure with mixed (**c**) standard (a = b) and (**d**) nonstandard (a ≠ b) unit cells, where a and b represent the lengths of black (L1_0_ phase) or white (L1_2_ phase) tiles and D characterizes the cell size. The yellow arrows represent the easy axis of L1_0_ phase.

The nanoarchitectonics of self-assembled nanostructures at the nanoscale has played a pivotal role in propelling the ongoing advancement of nanodevices in the current millennium. In particular, the intriguing quasiperiodic dual-phase chessboard-like nanostructure or nanochessboard appears to be a common phenomenon, as observed in various decomposing metal alloys [1,2,3,4,5,6,7,8]. Among these metal alloys, the Co-Pt nanochessboard represents a quasi-periodic nanocomposite comprising a magnetically hard L1_0_ phase and a magnetically soft L1_2_ phase [9,10,11,12,13,14]. Specifically, the L1_0_ phase has the primitive tetragonal (*tP*) Bravais lattice with the space group of P4/mmm, giving rise to a strong uniaxial magnetocrystalline anisotropy along the *c*-axis. The L1_2_ phase has the primitive cubic (*cP*) Bravais lattice with the space group of Pm$\bar{3}$m, associated with a relatively weak cubic magnetocrystalline anisotropy. The corresponding atomic structures are depicted in Figure 1a. Due to the high magnetocrystalline anisotropy of the hard phase and the large saturation magnetization of the soft phase, this nanocomposite exhibits both high coercivity and substantial magnetization remanence, making it a promising candidate for high-performance giant energy products in permanent magnets [15,16,17]. Since coercivity and magnetization are influenced by the magnetic domain structure, understanding the relationship between magnetic properties and domain structures is of significant importance.

The well-defined structure of the Co-Pt nanochessboard features a regular dispersion of the hard L1_0_ phase and the soft L1_2_ phase, and the short-range interaction between the two phases is a result of the exchange coupling effect occurring at the coherent interfaces that separate them. This exchange coupling is crucial for magnetic recording, spintronic technologies, and improved composite permanent magnets [18,19,20,21]. The periodic geometry and coherent heterophase interfaces in this unique nanochessboard structure offer an excellent platform to investigate the exchange coupling effect on magnetic properties of permanent magnets.

The Co-Pt composite materials can exhibit various nanochessboard structures depending on the processing conditions. In the direct eutectoid transformation, Al→L1_0_ + L1_2_, two types of nanochessboard structures can form: standard and nonstandard. Figure 1b schematically illustrates the typical nanochessboard structure with regular dispersion of the hard L1_0_ phase and the soft L1_2_ phase, which was observed by our prior experimental measurements [22,23]. Figure 1c illustrates the standard nanochessboard unit cell with equal edge length a = b, while Figure 1d presents the nonstandard nanochessboard unit cells with unequal edge lengths a ≠ b.

While previous theoretical and numerical studies have primarily focused on the ideal standard nanochessboard structure, the nonstandard nanochessboard has received little attention. For standard nanochessboards, the degree of exchange coupling has been found to vary with the tile size and periodicity of the chessboard structure [24]. In contrast, for nonstandard nanochessboards, the shape effect plays a significant role in addition to the influence of length scale on the degree of exchange coupling, setting it apart from the standard nanochessboard composite. Therefore, this work aims to investigate the exchange coupling effect on magnetic properties in nonstandard nanochessboards. To achieve this goal, we employed phase field modeling and computer simulations for a systematic study. The simulations revealed a dependence of the coercivity on both the tile size and shape, indicating an anisotropic exchange coupling in nonstandard nanochessboards.

## 2. Phase-Field Modeling

To simulate the magnetization evolution process in nanochessboard structures with varying tile size and shape, we employed the two-phase micromagnetic phase-field model. In this model, the two-phase Co-Pt nanochessboard structure is represented by a static phase-field variable θ(**r**), which takes a value of 1 for the tetragonal L1_0_ phase and 0 for the cubic L1_2_ phase. The structure of the magnetic domains in the nanochessboard is described by the magnetization vector field M(r), which is a function of the time-evolving unit vector m(r) and the saturation magnetization $M_{s}(r)$, depending on the characteristics of the two-phase nanostructure which is characterized by θ(r)

(1) $$M(r)=M_{s}(r)m(r)=[M_{s}^{L1_{0}}\theta (r)+M_{s}^{L1_{2}}(1-\theta (r))]m(r),$$

where $M_{s}^{L1_{0}}$ and $M_{s}^{L1_{2}}$ represent the saturation magnetization of the L1_0_ and the L1_2_ phases, respectively. The effective magnetic field $H^{eff}$ is determined by taking the functional derivative of the system’s free energy with respect to the magnetization vector field **M**

(2) $$H^{eff}=-1/\mu_{0}(\delta F/\delta M),$$

where $\mu_{0}$ denotes the vacuum permeability. The evolution of the magnetic domain structure is governed by the Landau–Lifshitz–Gilbert equation

(3) $$\dot{m}=\gamma H^{eff}\times m+\alpha m\times \dot{m},$$

where $\dot{m}$ represents the time derivative of the unit vector **m**, γ is the gyromagnetic ratio, and α is the damping parameter. The free energy of the two-phase Co-Pt magnetic system consists of the magnetocrystalline anisotropy energy, exchange energy, magnetostatic energy, and external magnetic energy (Zeeman energy). It can be formulated as [25]

(4) F=∫fan(m(r))d3r+A∫gradm(r) 2d3r+μ02∫d3k(2π)3n⋅M˜(k)2−μ0∫Hext⋅M(r)d3r

where *A* represents the exchange stiffness constant, and **H**^ext^ is the external magnetic field. The magnetocrystalline anisotropy energy density $f^{an}$ is dependent on the underlying two-phase microstructure variable θ(r) and the magnetization direction

(5) $$\begin{array}{cc} f^{\mathrm{an}}= & K_{1}^{L1_{0}}\left\{1-{\left[t(r)\cdot m(r)\right]}^{2}\;\right\}\theta (r)\\ + & K_{1}^{L1_{2}}\left[m_{1}^{2}(r)m_{2}^{2}(r)+m_{2}^{2}(r)m_{3}^{2}(r)+m_{3}^{2}(r)m_{1}^{2}(r)\right]\left(1-\theta (r)\right) \end{array}$$

where $K_{1}^{{L1}_{0}}$ and $K_{2}^{{L1}_{2}}$ represent the magnetocrystalline anisotropy constants of the L1_0_ and L1_2_ phases, respectively. The local orientation of the tetragonal axis of the L1_0_ phase, t(r), alternates between $\left[010\right]$ and [001] in neighboring L1_0_ tiles, as shown in Figure 1. The numerical solution of Equation (3) was accomplished through the utilization of our proprietary Fortran 90 code, employing a parallel algorithm facilitated by the Message Passing Interface (MPI). This code, developed within our research group and previously employed in other studies [22,24], was executed on the supercomputers at the Hefei Advanced Computing Center. The material parameters adopted in this study are [22]: $M_{{}_{S}}^{L1_{0}}$ = 4.2 × 10^5^ A/m, $K_{1}^{L1_{0}}$ = 1.5 × 10^6^ J/m^3^, $M_{{}_{S}}^{L1_{2}}$ = 5.2 × 10^5^ A/m, $K_{1}^{L1_{2}}$ = 2 × 10^4^ J/m^3^, and *A* = 2.5 × 10^−11^ J/m.

## 3. Results and Discussion

**Figure 2:**
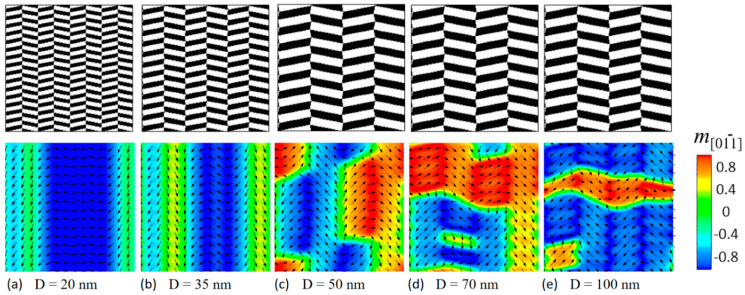
Phase morphologies and simulated initial magnetization distributions for nonstandard nanochessboards with the same aspect ratio of b/a = 4 but different unit cell sizes: (**a**) D = 20 nm, (**b**) D = 35 nm, (**c**) D = 50 nm, (**d**) D = 70 nm, and (**e**) D = 100 nm. Black arrows represent the in−plane magnetization components.

**Figure 3:**
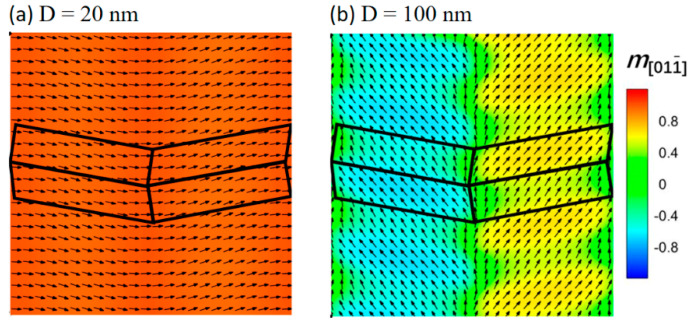
Magnetization distributions for [010]−poled nonstandard nanochessboards with unit cell sizes of (**a**) D = 20 nm and (**b**) D = 100 nm. Black solid lines depict the tile boundary in a unit cell. Black arrows represent the in−plane magnetization components.

In the chessboard structure, the L1_0_ tiles with high magnetic hardness are interconnected by the magnetically soft L1_2_ tiles in between. These L1_0_ tiles alternate between in-plane easy directions of $\left[010\right]$ and $\left[001\right]$, as illustrated in Figure 1. The strength of the exchange coupling is highly dependent on the tile size. To systematically investigate the exchange coupling effect in nonstandard nanochessboards, we considered the same aspect ratio but different cell sizes. Figure 2 displays the phase morphologies and phase-field simulated initial magnetization distributions for nonstandard nanochessboards with the same aspect ratio of b/a = 4 but varying cell sizes from D = 20 nm to 100 nm. Generally, as the cell size increases, the exchange coupling effect weakens. For relatively small unit cells, the strong exchange coupling causes significant deviations of magnetizations from the easy axis of the hard L1_0_ phase, resulting in the emergence of a new easy axis, as depicted in Figure 2a,b. On the other hand, for relatively large unit cells, the weakened exchange coupling only induces slight rotations of magnetizations around the easy axis of the hard L1_0_ phase, as shown in Figure 2c–e. To provide a clearer illustration of the magnetization behaviors, we simulated the magnetization distributions for $\left[010\right]$-poled nonstandard nanochessboards with two representative unit cell sizes of D = 20 nm and D = 100 nm, as presented in Figure 3.

According to Figure 3a with D = 20 nm, when the tile size is small enough for the exchange coupling effect to extend over them, the L1_2_ bridging tiles facilitate the exchange coupling of neighboring L1_0_ tiles. This type of coupling is referred to as hard–hard exchange coupling between neighboring L1_0_-L1_0_ tiles, in contrast to the hard–soft coupling observed between adjacent L1_0_-L1_2_ tiles. The hard–hard exchange coupling leads to a rotation of the local magnetization vectors away from the original $\left[001\right]$ or $\left[010\right]$ magnetic easy directions within individual L1_0_ tiles, towards the intermediate $\left[011\right]$ or $\left[01\bar{1}\right]$ direction. Consequently, the original individual [001] and [010] easy directions are weakened, while the new effective $\left[011\right]$ or $\left[01\bar{1}\right]$ easy direction in the nanocomposite system is strengthened. On the other hand, as shown in Figure 3b, when the cell size increases to D = 100 nm, the exchange coupling between hard magnetic regions notably decreases. As a result, the magnetizations within the L1_0_ hard magnetic tiles tend to align along their intrinsic easy axes. However, since the hard–soft exchange coupling between adjacent L1_0_-L1_2_ tiles still exists and is not negligible, the magnetizations in L1_2_ soft magnetic tiles remain almost collinear with the magnetizations in L1_0_ hard magnetic tiles. For a more detailed theoretical analysis of the hard–hard or hard–soft exchange coupling, one can refer to our prior work [24].

Assuming a uniaxial magnetocrystalline anisotropy in both the L1_0_ and L1_2_ phases, the critical exchange length can be approximated as $d_{cr}\sim 2\pi {\left(A_{soft}/2K_{hard}\right)}^{1/2}=18$ nm [15]. However, due to the unequal edge lengths of nonstandard nanochessboard tiles (a ≠ b), the exchange coupling becomes anisotropic. In cases where the unit cell size D remains the same but with b > a, the vertical ($\left[011\right]$) exchange coupling is more pronounced than the horizontal ($\left[01\bar{1}\right]$). Specifically, for nonstandard nanochessboard tiles with b = 4a, the critical cell size is approximately D_cr_~35 nm for the horizontal exchange coupling and D_cr_~141 nm for the vertical exchange coupling. It is worth noting that for standard nanochessboard tiles with a = b, the critical cell size is approximately D_cr_~35 nm for both horizontal and vertical exchange couplings, indicating a nearly isotropic exchange coupling effect.

Based on the analysis provided, the exchange coupling in nonstandard nanochessboards is influenced by both the tile size and shape, leading to different magnetic domain structures depending on the cell size. Let us revisit the magnetization distributions in Figure 2. For D = 20 nm, the cell size is below both the vertical and horizontal critical cell sizes, resulting in a strong hard–hard exchange coupling between neighboring L1_0_-L1_0_ tiles in both vertical and horizontal orientations. As a result, the magnetization vectors predominantly point towards vertical and horizontal directions, as the hard–hard exchange coupling promotes the effective $\left[01\bar{1}\right]$ and $\left[011\right]$ easy axes. The magnetizations tend to align along the effective easy axes rather than the original intrinsic easy axes. Figure 2a shows stripe “domains”, with the blue domain having magnetizations pointing in the $\left[01\bar{1}\right]$ direction and the green domain having magnetizations pointing in the $\left[0\bar{1}\bar{1}\right]$ direction. These stripe domains are vertical because the vertical exchange coupling is too strong to allow the appearance of 180° domain walls within these stripe domains. For D = 35 nm, although the cell size is increased, the exchange coupling is still strong enough to promote the effective easy axes, resulting in a similar domain structure (Figure 2b) as the case of D = 20 nm. As the cell size increases beyond the critical cell size for the horizontal exchange coupling, the nanocomposite enters the relatively weak hard-soft coupled regime and eventually the decoupled regime along the horizontal orientation. Consequently, the magnetizations tend to align along the original easy axes of the hard L1_0_ phase, and the domain walls are allowed to exist, as shown in Figure 2c–e. It is important to note that, since the critical cell size for the vertical exchange coupling is much larger than the horizontal, the vertical hard–hard exchange coupling still exists, unlike the negligible horizontal hard–hard exchange coupling. As a result, the magnetizations are almost collinear vertically but non-collinear horizontally, as depicted in Figure 2c–e.

**Figure 4:**
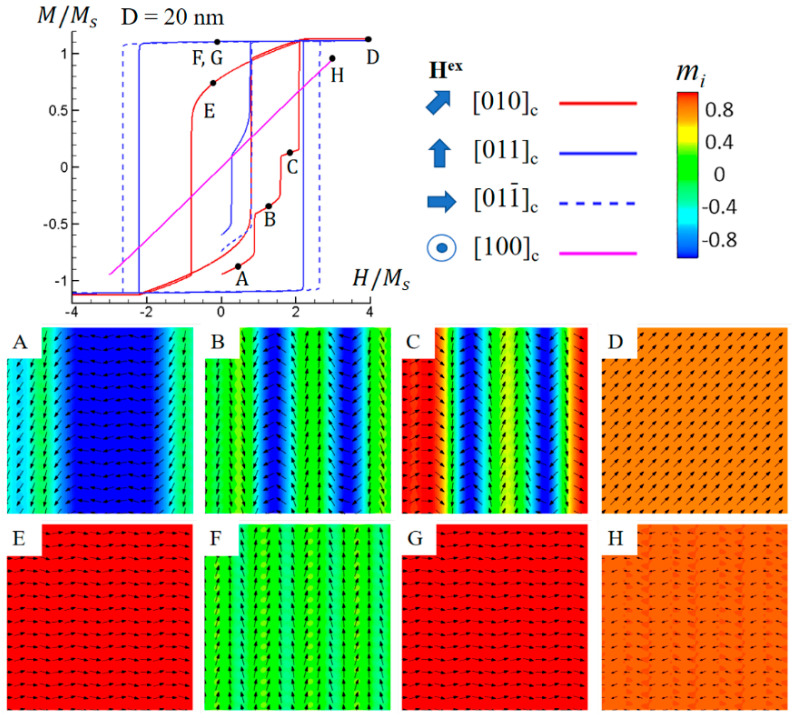
Simulated magnetic hysteresis loops and domain structures of Co−Pt nonstandard nanochessboard system with unit cell size of D = 20 nm under external magnetic field in $\left[010\right],[011]$, $\left[01\bar{1}\right]$, or [100] directions. Black arrows represent in−plane magnetization components and color contours the $\left[01\bar{1}\right]$ component for points (**A**–**G**), and the [100] component for point (**H**).

Commencing with the initial magnetic domain structures depicted in Figure 2 for the five cases, applying a magnetic field in different directions yields varying magnetic hysteresis loops and domain structure evolutions. Figure 4 shows simulated M-H hysteresis loops and domain structure transitions for the Co-Pt nonstandard nanochessboard system with a unit cell size of D = 20 nm under an external magnetic field in the $\left[010\right]$, [011], $\left[01\bar{1}\right]$, or [100] directions. Given that the cell size is considerably below the critical value, the strong hard–hard exchange coupling that extends over the neighboring hard–soft exchange coupling results in four effective easy directions: [011], $\left[01\bar{1}\right]$, $\left[0\bar{1}1\right]$, and $\left[0\bar{1}\bar{1}\right]$. Figure 4A displays the stripe “domain” pattern, where the magnetizations align along $\left[0\bar{1}1\right]$ and $\left[0\bar{1}\bar{1}\right]$. Under the application of a magnetic field in the [010] direction, the magnetizations undergo stepwise rotations and switching. Figure 4B indicates partial switching of $\left[0\bar{1}1\right]$ magnetizations to $\left[011\right]$, while Figure 4C shows complete switching of $\left[0\bar{1}\bar{1}\right]$ magnetizations to $\left[01\bar{1}\right]$. Figure 4D exhibits the saturated domain structure with magnetizations uniformly pointing in [010]. Upon unloading the magnetic field, all magnetizations revert to their easy axis, $\left[01\bar{1}\right]$, forming the remanent domain structure, as depicted in Figure 4E. Throughout this domain evolution process, the magnetization rotation mechanism takes precedence over the domain wall motion mechanism. Figure 4F,G show the remanent domain structures following magnetic polarization in the [011] and $\left[01\bar{1}\right]$ directions, respectively. As expected, magnetizations align homogeneously vertically and horizontally after polarization in the promoted effective easy directions. Figure 4H illustrates magnetization distribution under a substantial magnetic field in the [100] direction. The magnitude of the in-plane magnetizations is notably small, indicating that the magnetization is predominantly achieved by the out-of-plane field [100]. This outcome is attributed to the reduction in anisotropy due to the hard–hard exchange coupling in the plane, facilitating easier magnetization rotation. Upon field release, magnetizations eventually homogeneously align in the effective easy direction $\left[01\bar{1}\right]$. Unlike the hard L1_0_ phase, which only features in-plane easy axes, the soft L1_2_ phase also possesses an out-of-plane easy axis [100]. Due to the weaker anisotropy of the L1_2_ phase and the strong hard–soft exchange coupling, the out-of-plane component of L1_2_ phase magnetizations cannot be maintained upon field unloading. In other words, domain evolution is reversible in the loading–unloading process. As a result, the hysteresis phenomenon is nearly absent.

**Figure 5:**
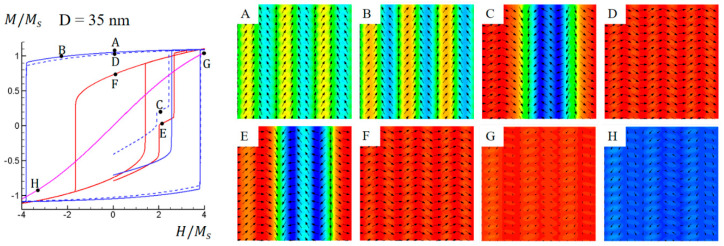
Simulated magnetic hysteresis loops and domain structures of Co−Pt nonstandard nanochessboard system with unit cell size of D = 35 nm under external magnetic field in $\left[010\right],[011]$, $\left[01\bar{1}\right]$ or [100] direction. Refer to Figure 4 for the meaning of colored lines. Black arrows represent in−plane magnetization components and color contours the $\left[01\bar{1}\right]$−component for points (**A**–**F**) while [100]−component for points (**G**,**H**).

Figure 5 illustrates simulated M-H hysteresis loops and domain structure evolutions for the Co-Pt nonstandard nanochessboard system with a unit cell size of D = 35 nm under external magnetic fields in the $\left[010\right]$, [011], $\left[01\bar{1}\right]$, or [100] directions, with the initial domain structure as shown in Figure 2b. The hysteresis loops and domain evolution are similar to the case of D = 20 nm, as shown in Figure 4. Figure 5A–D show the domain structure evolution under vertical and horizontal magnetic fields. Since the applied magnetic fields are parallel to the effective easy directions, these two hysteresis loops are wider than the others. As the cell size increases from 20 nm to 35 nm, the magnetization homogeneity of the remanent domain structures is reduced due to the weakened exchange coupling, as shown in Figure 5A,D. Figure 5E,F illustrate the domain structure evolution under magnetic field in the $\left[010\right]$ direction, while Figure 5G,H illustrate the domain structure evolution under magnetic field in the $\left[100\right]$ direction. Due to the strong hard–soft exchange coupling, the out-of-plane component of L1_2_ phase magnetizations still cannot be maintained if the field is unloaded. This is also indicated by the lack of hysteresis feature in the M-H loop with the field along the $\left[100\right]$ direction. Additionally, the curve is not as linear as in the case of D = 20 nm, indicating a reduction in exchange coupling as the cell size is increased. It is worth noting that almost all the hysteresis loops become wider as the cell size increases, which is caused by the weakening of the hard–hard exchange coupling. In comparison with the intrinsic easy axis and magnetocrystalline anisotropy energy of the hard L1_0_ phase, the effective easy axis is associated with a lower anisotropy energy and hence a smaller coercivity. Thus, the weakening of exchange coupling results in an enhanced anisotropy energy and, consequently, an increased coercivity.

**Figure 6:**
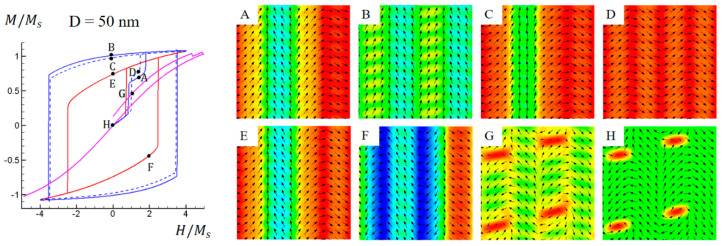
Simulated magnetic hysteresis loops and domain structures of Co−Pt nonstandard nanochessboard system with unit cell size of D = 50 nm under external magnetic field in $\left[010\right],[011]$, $\left[01\bar{1}\right]$, or [100] directions. Refer to Figure 4 for the meaning of colored lines. Black arrows represent in−plane magnetization components and color contours the $\left[01\bar{1}\right]$ component for points (**A**–**F**), and the [100] component for points (**G**,**H**).

Figure 6 illustrates simulated M-H hysteresis loops and domain structure evolutions for the Co-Pt nonstandard nanochessboard system with a unit cell size of D = 50 nm under external magnetic fields in the $\left[010\right]$, [011], $\left[01\bar{1}\right]$ or [100] direction, with the initial domain structure as shown in Figure 2c. As the cell size is beyond the critical value, the horizontal hard-soft exchange coupling is decoupled, resulting in magnetizations in the hard L1_0_ phase tending to align with their original easy axes. Figure 6A–F depict the domain structure evolution under in-plane magnetic fields. Unlike the previous two cases, where only the magnetization rotation mechanism dominated, in this case, the domain wall motion mechanism also plays a crucial role in the domain evolution process. Figure 6G,H show the domain structure under out-of-plane magnetic fields. Due to the reduced exchange coupling, nonzero out-of-plane components are allowed to exist. In this situation, the out-of-plane magnetizations appear in the form of vortex or antivortex, which are domain walls separating vertical domains.

**Figure 7:**
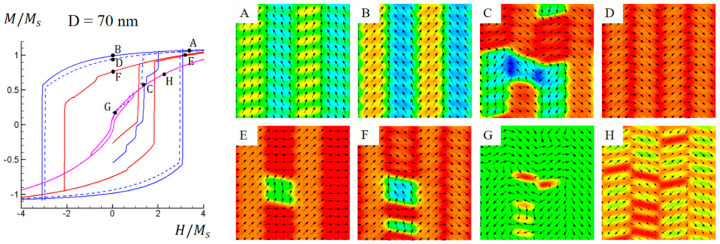
Simulated magnetic hysteresis loops and domain structures of Co−Pt nonstandard nanochessboard system with unit cell size of D = 70 nm under external magnetic field in $\left[010\right],[011]$, $\left[01\bar{1}\right]$, or [100] directions. Refer to Figure 4 for the meaning of colored lines. Black arrows represent in-plane magnetization components and color contours the $\left[01\bar{1}\right]$ component for points (**A**–**F**), and the [100] component for points (**G**,**H**).

As the cell size further increases, the exchange coupling becomes weaker, allowing more domains or domain walls to appear in the initial domain structure, as shown in Figure 2d. Figure 7 shows simulated M-H hysteresis loops and domain structure evolutions for the Co-Pt nonstandard nanochessboard system with a unit cell size of D = 70 nm under external magnetic fields in four different directions. In this hard–soft coupling regime, the effect of exchange coupling weakens with increasing cell size, leading to a reduction in the width of hysteresis loops under in-plane fields, indicating a diminished coercivity. Figure 7A–F illustrate the domain structure evolution under in-plane magnetic fields. Although the horizontal exchange coupling is weakened, the vertical coupling remains strong enough. Consequently, the magnetizations tend to stay collinear vertically while remaining non-collinear horizontally. Figure 7G,H show the domain structures under out-of-plane magnetic fields. Due to the strong in-plane anisotropy of the hard L1_0_ phase, magnetizations prefer to stay in-plane to accommodate the underlying magnetic easy directions. The soft L1_2_ phase possesses a lower anisotropy, and the magnetizations are more prone to stay in the out-of-plane easy axis. However, only the magnetizations within domain walls can maintain the out-of-plane state with an unloaded field, since the exchange coupling effect from vertical neighboring L1_0_ phases encourages most magnetizations to stay in-plane. Under the out-of-plane magnetic field, these out-of-plane magnetizations can be oriented either along the [100] or $\left[\bar{1}00\right]$ direction, which eventually results in the hysteresis characteristic.

**Figure 8:**
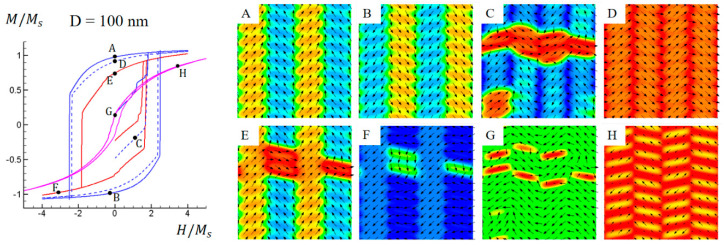
Simulated magnetic hysteresis loops and domain structures of Co−Pt nonstandard nanochessboard system with unit cell size of D = 100 nm under external magnetic field in $\left[010\right],[011]$, $\left[01\bar{1}\right]$, or [100] directions. Refer to Figure 4 for the meaning of colored lines. Black arrows represent in−plane magnetization components and color contours the $\left[01\bar{1}\right]$ component for points (**A**–**F**), and the [100] component for points (**G**,**H**).

As the unit cell size D increases from 70 nm to 100 nm, the exchange coupling effect becomes weaker. Consequently, the width of hysteresis loops under in-plane fields and hence the coercivity are further reduced, as depicted in Figure 8. Figure 8A–F illustrate the domain structure evolution under in-plane magnetic fields. The vertical exchange coupling is weakened compared to previous cases, resulting in reduced magnetization collinearity between L1_0_ and L1_2_ phases and making the difference between the two phases more apparent. Moreover, the reduced exchange coupling also allows more out-of-plane magnetizations to be maintained, as shown in Figure 8G,H. As a result, the M-H hysteresis characteristic with an out-of-plane field is further enhanced, leading to a higher coercivity.

**Figure 9:**
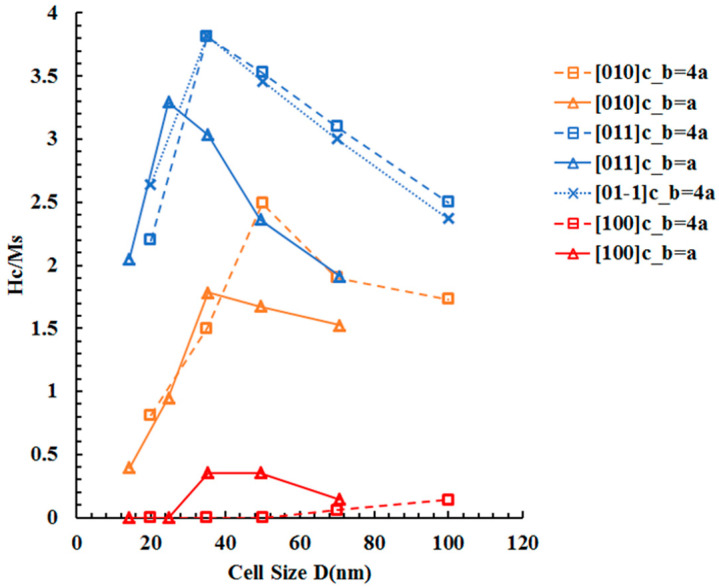
Simulated magnetic coercive field as a function of unit cell size D for both standard (b = a, solid lines) and nonstandard (b = 4a, dashed lines) Co-Pt nanochessboards under external magnetic field in $\left[010\right],[011]$, $\left[01\bar{1}\right]$, or [100] directions.

The simulated hysteresis loops in Figure 4, Figure 5, Figure 6, Figure 7 and Figure 8 demonstrate that the magnetic coercive field has a strong dependency on the cell size D. To further examine the exchange coupling effect on magnetic coercivity, we plotted the simulated magnetic coercive field as a function of unit cell size D for both standard (b = a) and nonstandard (b = 4a) Co-Pt nanochessboards under external magnetic fields in different directions, as shown in Figure 9. Firstly, both standard and nonstandard nanochessboards exhibit similar behaviors of coercivity under in-plane magnetic fields. As the cell size D increases, the coercivity initially increases and then decreases. This universal behavior can be explained by considering the magnetization rotation path under the magnetic field, which depends on the tile size. For relatively small tile sizes, the magnetization rotation follows Path I, where the hard–hard exchange coupling is so strong that the magnetizations of neighboring hard L1_0_ phases are bonded together. This bonding enables easy rigid-body rotation against the original magnetocrystalline anisotropy energy. The smaller the tile size, the stronger the binding effect. Consequently, as the tile size decreases, the effective anisotropy is reduced, leading to a smaller coercivity. For relatively large tile sizes, the magnetization rotation follows Path II, where the hard–hard exchange coupling is weak enough to allow the magnetizations of neighboring hard L1_0_ phases to rotate separately. The larger the tile size, the easier the separate rotation. Therefore, as the tile size increases, the splitting energy is reduced, resulting in a smaller coercivity. The magnetic coercivity behavior observed in Co-Pt nanochessboards with varying tile sizes is consistent with the corresponding experimental measurements [23].

Secondly, compared to the standard nanochessboards, the coercivity peaks of nonstandard nanochessboards were found to shift to the right (toward a higher D). This can be explained by the anisotropic exchange coupling effect. For the same cell size D, the horizontal exchange coupling is generally the same for both standard and nonstandard nanochessboards. However, the nonstandard case exhibits a stronger vertical exchange coupling than the standard case, meaning a larger cell size D is required to diminish the stronger exchange coupling of nonstandard nanochessboards. This results in a generally enhanced coercivity magnitude for nonstandard nanochessboards. Alternatively, this shift in coercivity peak can also be explained by considering the surface-area-to-volume ratio of nanochessboard phases. For this nanorod structure, the surface-area-to-volume ratio is directly related to the aspect ratio of the tiles. Since nonstandard nanochessboards have a larger aspect ratio (b/a = 4), their corresponding surface-area-to-volume ratio is also larger than the standard case. A larger surface-area-to-volume ratio is usually associated with a stronger exchange coupling effect, thus leading to a rightward shift of the coercivity peak.

## 4. Conclusions

The binary Co-Pt nanochessboard system is composed of a magnetically hard tetragonal L1_0_ phase and a magnetically soft cubic L1_2_ phase. This unique nanochessboard structure with periodic geometry and coherent heterophase interfaces provides an excellent platform for investigating the exchange coupling effect on magnetic properties of permanent magnets. During the direct eutectoid transformation, two types of nanochessboard structures can form: standard and nonstandard. The standard nanochessboard has equal edge lengths (a = b), while the nonstandard nanochessboard has unequal edge lengths (a ≠ b). As an exchange-coupled magnetic nanocomposite, Co-Pt nanochessboards with nonstandard tiles exhibit a distinct exchange coupling effect on magnetic domain structures and coercivity compared to the standard tiles. In this study, we employed phase-field modeling and computer simulation to systematically explore the exchange coupling effect on magnetic properties in nonstandard nanochessboards with a tile aspect ratio of b/a = 4. The simulations revealed a dependence of magnetic coercivity on the tile size, wherein an initial magnetic hardening occurred below the critical exchange length, followed by magnetic softening above the critical exchange length, similar to the behavior observed in standard nanochessboards. Additionally, the unequal edge lengths led to anisotropic exchange coupling. For the same unit cell size D, the surface-area-to-volume ratio of nonstandard nanochessboards was larger than that of the standard case, resulting in a stronger exchange coupling effect and causing a rightward shift of the coercivity peak. These findings provide new insights into the exchange coupling effect on the magnetic properties of nonstandard Co-Pt nanochessboards and offer guidance for experimental strategies to modulate magnetic properties in Co-Pt nanochessboard systems.

## Data Availability

Not applicable.
